# AGBL4 promotes malignant progression of glioblastoma via modulation of MMP-1 and inflammatory pathways

**DOI:** 10.3389/fimmu.2024.1420182

**Published:** 2024-06-28

**Authors:** Shuai Zhang, Lilin Cheng, Yandong Su, Zhongrun Qian, Zhen Wang, Chao Chen, Rong Li, Aikang Zhang, Jiawei He, Jiangxin Mao, Hongxiang Wang, Juxiang Chen

**Affiliations:** ^1^ Department of Neurosurgery, Changhai Hospital, Naval Medical University, Shanghai, China; ^2^ Department of Neurosurgery, The First Affiliated Hospital of University of Science and Technology of China, Division of Life Sciences and Medicine, Hefei, Anhui, China

**Keywords:** glioblastoma, prognosis, AGBL4, MMP-1, single-cell sequencing

## Abstract

**Introduction:**

Glioblastoma multiforme (GBM), the most common primary malignant brain tumor, is notorious for its aggressive growth and dismal prognosis. This study aimed to elucidate the molecular underpinnings of GBM, particularly focusing on the role of AGBL4 and its connection to inflammatory pathways, to discover viable therapeutic targets.

**Methods:**

Single-cell sequencing was utilized to examine the expression levels of AGBL4 and functional assays were performed to assess the effects of AGBL4 modulation.

**Results:**

Our findings identified the significant upregulation of AGBL4 in GBM, which correlated with adverse clinical outcomes. Functional assays demonstrated that AGBL4 knockdown inhibited GBM cell proliferation, migration, and invasion and influenced inflammatory response pathways, while AGBL4 overexpression promoted these activities. Further investigation revealed that AGBL4 exerted its oncogenic effects through modulation of MMP-1, establishing a novel regulatory axis critical for GBM progression and inflammation.

**Discussion:**

Both AGBL4 and MMP-1 may be pivotal molecular targets, offering new avenues for targeted therapy in GBM management.

## 1 Introduction

Gliomas are the most common malignant primary tumors in the central nervous system, derived from glial or precursor cells and encompass diverse histopathological subtypes, including GBM, astrocytoma, oligodendroglioma, ependymoma, and oligoastrocytoma. GBM is the most common and aggressive form, making up the majority of cases (1). Despite standard treatment involving maximal safe resection, chemotherapy, radiotherapy, and tumor treating fields, GBM remains therapeutically challenging due to its aggressive nature, tendency to infiltrate the surrounding brain, and develop resistance to therapies (2). This results in a dismal 5-year survival rate of merely 4.7% (3). Consequently, there is an urgent need to elucidate the molecular underpinnings of GBM to improve diagnostic efficacy and develop novel targeted therapies (4).

The advent of single-cell sequencing technology has revolutionized our understanding of cellular processes in biology (5). By enabling the measurement of individual cell genomes, single-cell sequencing facilitates the analysis of differentially expressed genes (DEGs), the identification of key factors dysregulated during tumorigenesis, and the construction of regulatory network and clonality trees within tumor lesions. It also enables the study of tumor heterogeneity across multiple levels, which is crucial for understanding resistance to therapy and for creating new treatment approaches (6). Therefore, single-cell sequencing has been widely employed for detecting mutations and studying the epigenomic changes during tumor progression.

Emerging evidence implicates the ATP/GTP-binding protein-like 4 gene (AGBL4) in various pathological processes, including antituberculosis drug-induced hepatotoxicity (7), cardiometabolic risk (8), and colorectal cancer, where it is anticipated to serve as a novel biomarker (9). However, its role in gliomas, particularly GBM, remains largely unexplored. In this study, we employed single-cell sequencing to confirm high expression levels of AGBL4 in GBM tissues linked to poor outcomes, supported by data from The Cancer Genome Atlas (TCGA) and Changhai Hospital. Functional assays demonstrated its capacity to promote GBM cell proliferation, migration, and invasion. Subsequent investigations identified matrix metalloproteinase-1 (MMP-1) as a key gene increased in GBM tissues and a likely target of AGBL4. Reducing AGBL4 levels significantly hindered GBM growth in xenograft models, a process that MMP-1 could reverse.

Further analysis indicated that AGBL4-related DEGs like MMP-1, Fos proto-oncogene (FOS), and FosB proto-oncogene (FOSB) are involved in the interleukin (IL)-17 signaling pathway, suggesting that AGBL4 and MMP-1 could influence GBM progression via inflammatory pathways. Subsequent analyses showed a complex relationship among AGBL4, MMP-1, and other inflammatory genes in regulating the GBM tumor microenvironment, affecting tumor behavior and patient survival. These findings highlight the potential of inflammation-related factors as focal points for future research and the development of novel therapeutic strategies for GBM.

## 2 Materials and methods

### 2.1 Patients and tissue samples

Specimen collection and clinical data were approved by the Research Ethics Committee of Changhai Hospital, Naval Medical University. Written informed consent was secured from each participant. The study included three primary and three recurrent GBM samples from six Chinese patients for single-cell sequencing. Additionally, eight fresh GBM samples and four normal brain tissues from traumatic injury patients were obtained. Sixty-five paraffin-embedded primary GBM specimens from January 2005 to December 2019, with clinical data and follow-up, were analyzed. GBM patient datasets from TCGA database provided external validation.

### 2.2 Single-cell sequencing

GBM sample single-cell sequencing libraries were constructed following the Chromium Next GEM Single Cell 3’ Reagent Kits v3.1. Gene expression matrices were generated and processed using Cell Ranger software on the 10×Genomics platform. Genomic and transcriptomic mapping was done using Spliced Trans Alignment to a Reference software, producing gene counts matrices per cell. Cell filtration, standardization, classification, differential gene expression analysis, and marker gene screening were conducted using the Seurat package in R studio. Sequencing was outsourced to Oebiotech Co., Ltd., Shanghai, China.

### 2.3 Western blot analysis

Samples were lysed using RIPA buffer (cat R0010, Solarbio, Beijing, China) with protease inhibitors (SKU 11836153001, Roche, Basel, Switzerland). Proteins were separated on 10% SDS-PAGE gels (cat 20325ES62, Yeason, Shanghai, China) and transferred onto PVDF membranes (cat GVWP02500, Millipore, MA, USA). Membranes were incubated with anti-human AGBL4 antibody (1:1000) and anti-actin antibody (1:10,000) overnight at 4°C, then with secondary antibodies for 1 hour at room temperature. Protein bands were visualized using an ECL kit (cat PI32209, Thermo Scientific Pierce, Waltham, MA, USA).

### 2.4 Quantitative real-time PCR

Total RNA was extracted with TRIzol ^®^ reagent (cat 15596026CN, Thermo Fisher Scientific, Waltham, MA, USA). Complementary DNA was synthesized using HiScript II RT SuperMix (cat R223–01, Vazyme, Nanjing, China). RT-PCR quantified AGBL4 mRNA levels using GAPDH as an endogenous control with primers Human-AGBL4-F (AATCTACCAGCAGACCAAAATG) and Human-AGBL4-R (TCAAAACAAAAGGCAAAGGAC).

### 2.5 Cell culture and transfection

GBM cell lines T98G, U251-MG, U87-MG, and A172, sourced from the Cell Bank of Chinese Academy of Science, were maintained in Dulbecco’s Modified Eagle’s Medium supplemented with 10% fetal bovine serum at 37°C in a 5% CO_2_ atmosphere. Lentiviral vectors for AGBL4 knockdown (KD) and overexpression (OE) were produced by Hanyin Biotech, Shanghai, China. Specific AGBL4-KD and AGBL4-OE lentiviruses were used to transduce U87-MG and A172, and T98G and U251-MG cell lines, respectively. The sequences for AGBL4-shRNAs were: shRNA1: GAGGGAATGTGAGCAAATA, shRNA2: CCGGACCATAGGAAGAACT, shRNA3: GCTTACTGCTACCCATATA.

### 2.6 Cell viability, colony formation, scratch assay, and Matrigel-transwell assay

Cell viability was assessed using the Cell Counting Kit-8 (CCK-8, cat CK04–01, Dojindo, Japan) by measuring the optical density at 450 nm at 24, 48, 72, 96, and 120 hours post-treatment. Colony formation efficiency was evaluated by seeding cells in 6-well plates and staining emerging colonies with 0.1% crystal violet. To assess cell migration, a scratch assay was performed. Cells were grown in 6-well plates and a scratch was made in the center of the wells using a 200 µL pipette tip. After washing away the cellular debris and further incubating, images of the scratch were captured to evaluate the migration rate by measuring the gap closure. For invasion assays, the upper chamber of a transwell apparatus was coated with Matrigel (cat CLS3422, 8-µm pores, Millipore, MA, US) and seeded with 5 × 10^4^ cells in 100 μL of serum-free medium. The lower chamber was filled with 600 µL of complete culture medium. After overnight incubation, cells that migrated to the underside of the membrane were stained with 0.1% crystal violet, and five random fields were counted under a light microscope.

### 2.7 Immunohistochemical analysis

Immunohistochemistry was performed to detect AGBL4 expression in a GBM tissue microarray with 65 samples from the Department of Neurosurgery, Changhai hospital, Naval Medical University. The procedure included fixing, dehydrating, embedding, and sectioning tissues, which were then deparaffinized and rehydrated. Heat-mediated antigen retrieval was performed, followed by blocking of endogenous peroxidase and nonspecific binding. Sections were incubated with primary and secondary antibodies, developed with chromogen, counterstained with hematoxylin, and finally, dehydrated, cleared, and mounted for microscopic examination. The percentage of positive cells was divided into 0 (0–5%), 1 (6–25%), 2 (26–50%), 3 (51–75%) and 4 (76–100%). The intensity of protein expression was determined as 0 (no staining), 1 (weakly staining), 2 (moderately staining) and 3 (strongly staining). The scores was calculated by multiplying the percentage of positive cells and the intensity of protein expression as follows: 0 (-), 2–3 (+), 4–6 (++), and >6 (+++). A total score of ≥4 points categorizes the specimens into the high AGBL4 group, while scores <4 points indicate low AGBL4 group.

### 2.8 Xenograft animal model

Male athymic nu/nu mice aged 6 weeks, obtained from Shanghai Jiao Tong University, were used in compliance with guidelines set by the Institutional Animal Care and Use Committee of Changhai Hospital, Naval Medical University. For tumor induction, we used three groups of mice (6 mice per group) injected with different cell lines: U87-MG control cells (U87MG-NC), AGBL4-knockdown U87-MG cells (U87MG-AGBL4-KD), and U87-MG cells with both AGBL4 knockdown and MMP-1 overexpression (U87MG-AGBL4-KD+MMP1-OE). Each mouse was anesthetized and their heads were secured in a stereotaxic instrument for precise intracranial injection of 5 × 10^5^ cells into the corpus striatum. Post-injection, the mice were monitored every three days for changes in behavior and body weight. Magnetic resonance imaging (MRI) was utilized to assess tumor development when clinical signs such as reduced eating, decreased movement, circling behavior, or weight loss were observed. Tumor volumes were calculated based on the MRI data, and body weight differences among the three groups were compared on the day of MRI scanning. Mice were euthanized at humane endpoints, which were clearly defined by severe neurological dysfunction, inability to access food or water, unrelieved pain, or other signs indicating a severe decline in quality of life. The overall survival periods were recorded and the brains were harvested for further histopathological examination.

### 2.9 Statistics

Statistical analyses were performed using SPSS software (version 19.0). Student’s t-test was used to compare the mean differences between two groups. Kaplan-Meier survival analysis and log-rank test were employed to evaluate the survival outcomes among different groups. All statistical analyses were two-sided, and P < 0.05 was considered statistically significant. Statistical graphs were drawn using GraphPad Prism 7 software (GraphPad Software Inc., San Diego, CA, USA).

Methods for Hematoxylin-Eosin (H&E) staining and bioinformatics analysis are detailed in the **Supplementary Materials**.

## 3 Results

### 3.1 AGBL4 is highly expressed in GBM and predicts poor prognosis

**Figure 1:**
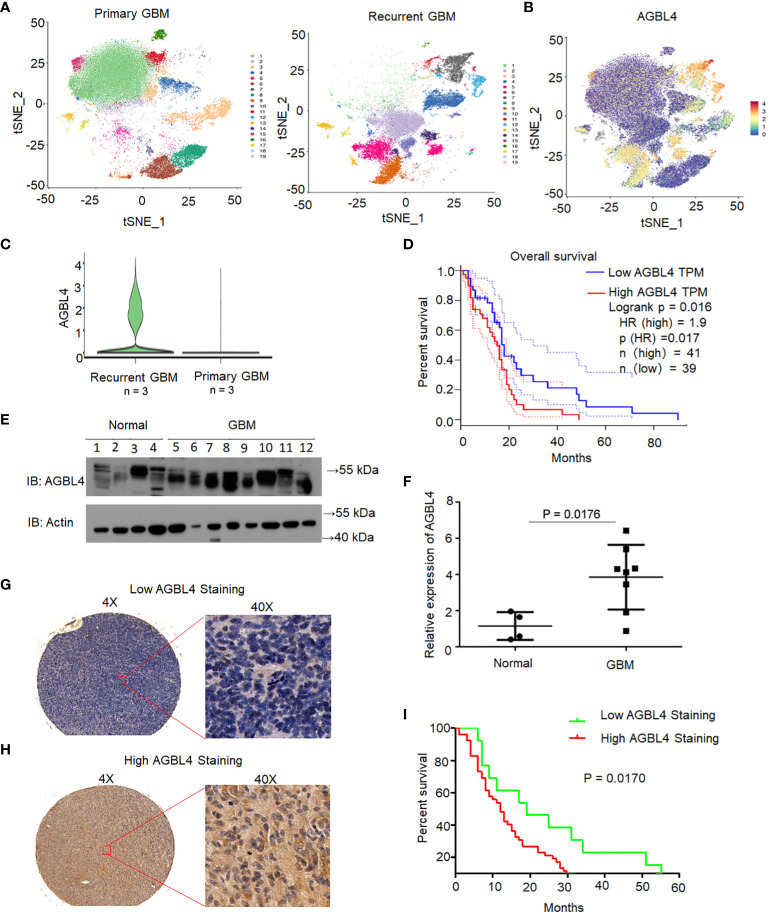
AGBL4 was highly expressed in GBM and predicted poor prognosis. **(A)** t-SNE visualization of 19 distinct clusters identified from single-cell RNA sequencing of primary and recurrent GBM samples. **(B)** Expression of AGBL4 across the clusters revealed by t-SNE plot. **(C)** AGBL4 expression is significantly higher in recurrent GBM compared to primary GBM samples. **(D)** Survival curves of GBM patients with low AGBL4 or high AGBL4 expression, obtained from TCGA database, P=0.017. **(E)** WB analysis confirms elevated AGBL4 protein levels in GBM tissues compared to normal brain samples. **(F)** Quantification of qRT-PCR verifies the upregulation of AGBL4 in GBM relative to normal brain tissues, P=0.0176. **(G, H)** Representative images of immunohistochemical staining show **(G)** low and **(H)** high AGBL4 expression in GBM tissues. Scale bars: 100 μm (4X), 25 μm (40X). **(I)** Kaplan-Meier analysis demonstrates that GBM patients with high AGBL4 staining have significantly shorter survival times compared to those with low AGBL4 expression, P = 0.0170.

Single-cell sequencing was performed on both primary and recurrent GBM specimens. Dimensionality reduction via the t-distributed stochastic neighbor embedding (t-SNE) algorithm revealed nineteen distinct clusters (**Figure 1A**). AGBL4 expression was observed across a majority of these tumor clusters (**Figure 1B**), with a significant upregulation in recurrent GBM compared to primary GBM (**Figure 1C**). Survival analysis using TCGA database indicated that elevated AGBL4 levels were associated with a worse prognosis in GBM patients (**Figure 1D**).

To validate the role of AGBL4 in GBM prognosis, we analyzed AGBL4 expression in normal brain tissues (n=4) and GBM tissues (n=8) through RT-PCR and WB. The WB results confirmed a marked increase in AGBL4 levels in GBM tissues relative to normal brain samples (**Figures 1E, F**). Immunohistochemical analysis was conducted on primary GBM tissue microarray. Based on the scoring criteria outlined before, samples were classified into low and high AGBL4 expression groups. Representative images of low (**Figure 1G**) and high (**Figure 1H**) AGBL4 groups illustrate the distinctions in staining intensity and cellular distribution. Survival analysis demonstrated a significant association between AGBL4 expression levels and patient outcomes. Specifically, patients categorized into the high AGBL4 group (scores ≥4) exhibited notably shorter survival times compared to those in the low AGBL4 group (scores <4) (P=0.017) (**Figure 1I**).

Altogether, these results demonstrate that AGBL4 expression is significantly elevated in GBM and its overexpression is predictive of poor prognosis in both our cohort and TCGA dataset.

### 3.2 Knockdown of AGBL4 inhibits GBM cell proliferation, migration, and invasion

**Figure 2:**
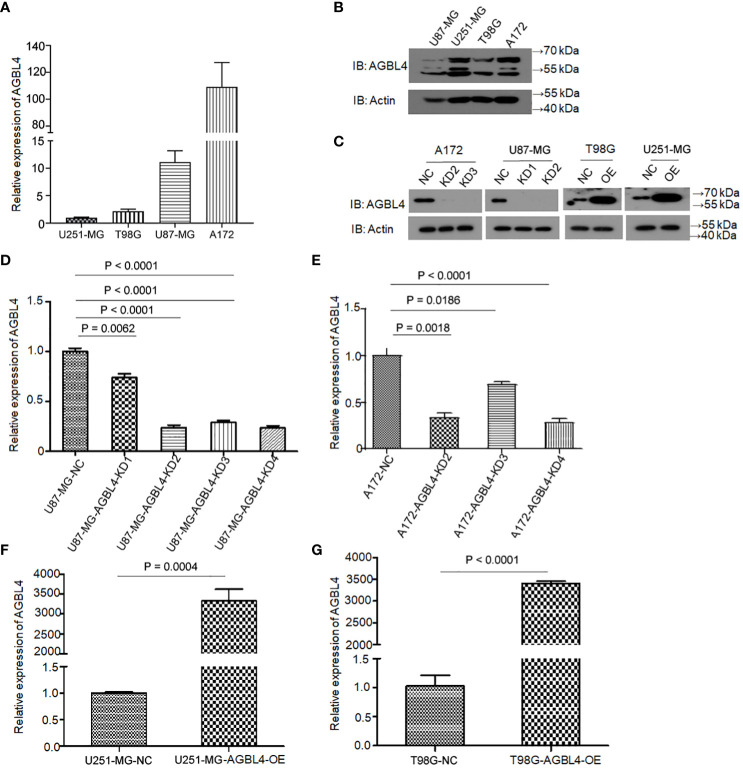
Relative expression levels of AGBL4 in GBM cells. **(A)** QRT-PCR analysis shows varying expression levels of AGBL4 across different GBM cell lines, with U87-MG and A172 exhibiting higher expression compared to T98G and U251-MG. **(B, C)** WB analysis confirms the protein expression patterns of AGBL4 in **(B)** U87-MG, U251-MG, T98G, and A172 cell lines and **(C)** after AGBL4 knockdown in A172 and U87-MG cells, and overexpression in T98G and U251-MG cells. **(D–G)** Quantification of qRT-PCR demonstrates successful AGBL4-KD in **(D)** U87-MG and **(E)** A172 cells, and successful AGBL4-OE in **(F)** U251-MG and **(G)** T98G cells.

To determine the roles of AGBL4 in GBM cell functions, we first analyzed AGBL4 expression in various GBM cell lines. Using the 2^-ΔΔCt^ method, RT-PCR results showed differential expression levels of AGBL4, with U87-MG and A172 cells exhibiting higher expression compared to T98G and U251-MG cells (**Figure 2A**). Additionally, WB analysis confirmed these findings, showing protein expression levels consistent with the RT-PCR results (**Figure 2B**). Following the knockdown of AGBL4 using the most effective shRNA sequence (shRNA2: CCGGACCATAGGAAGAACT) in U87-MG and A172 cell lines, WB analysis confirmed the efficient reduction of AGBL4 expression The knockdown efficiency was quantified at approximately 70% in the U87-MG cell line and around 65% in the A172 cell line (**Figures 2C–E**).

**Figure 3:**
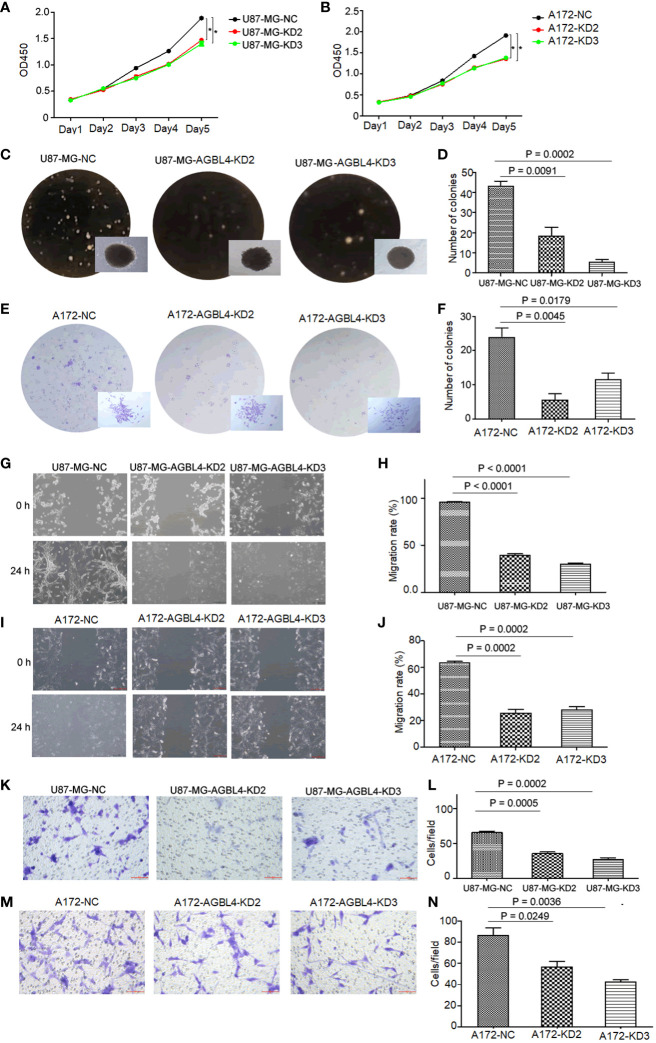
Knockdown of AGBL4 inhibits GBM cell proliferation, migration and invasion abilities. **(A, B)** CCK-8 assays show reduced proliferation in **(A)** U87-MG and **(B)** A172 cells after AGBL4 knockdown. **(C–F)** Colony formation assays demonstrate decreased colony numbers and sizes in **(C, D)** U87-MG and **(E, F)** A172 cells after AGBL4 knockdown. **(G–J)** Scratch migration assays reveal impaired migratory ability of **(G, H)** U87-MG and **(I, J)** A172 cells following AGBL4 knockdown. **(K–N)** Matrigel transwell invasion assays illustrate diminished invasive potential in **(K, L)** U87-MG and **(M, N)** A172 cells upon AGBL4 knockdown.

Functional assays were then performed to investigate the effect of AGBL4 on GBM cell pathology. The CCK-8 assay and colony formation assay, both indicative of cell proliferative capacity, showed that AGBL4 knockdown significantly decreased proliferation in U87-MG and A172 cells (**Figures 3A–F**). Scratch assays demonstrated that AGBL4 knockdown also decreased the migratory capabilities of these cells (**Figures 3G–J**). Finally, Matrigel-transwell assays provided quantitative and visual evidence of the diminished invasion capacity following AGBL4 knockdown (**Figures 3K–N**).

These findings collectively suggest that AGBL4 is integral to the proliferative, migratory, and invasive characteristics of GBM cells, confirming its potential as a target for GBM therapy.

### 3.3 Overexpression of AGBL4 improves GBM cell proliferation, migration and invasion

**Figure 4:**
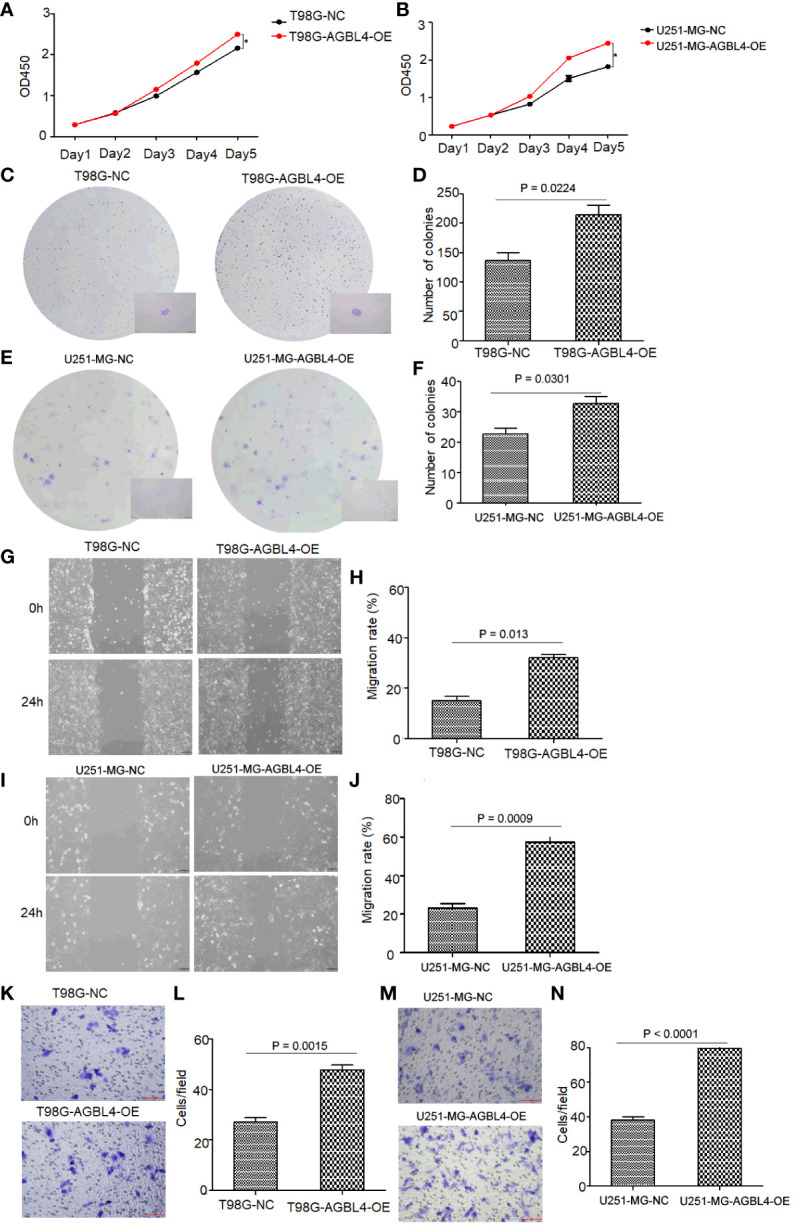
High expression of AGBL4 improves GBM cell proliferation, migration and invasion abilities. **(A, B)** CCK-8 assays demonstrate enhanced proliferation in **(A)** T98G and **(B)** U251-MG cells after AGBL4 overexpression. **(C–F)** Colony formation assays show increased colony numbers and sizes in **(C, D)** T98G and **(E, F)** U251-MG cells after AGBL4 overexpression. **(G–J)** Scratch migration assays indicate improved migratory ability of **(G, H)** T98G and **(I, J)** U251-MG cells following AGBL4 overexpression. **(K–N)** Matrigel transwell invasion assays reveal elevated invasive potential in **(K, L)** T98G and **(M, N)** U251-MG cells upon AGBL4 overexpression.

To investigate the roles of AGBL4 in GBM growth, we overexpressed AGBL4 in T98G and U251-MG cells. WB results verified the overexpression of AGBL4 in these cells (**Figure 2C**). RT-PCR also confirmed that the relative expression levels of AGBL4 were significantly increased in both U251-MG and T98G overexpression groups (**Figures 2F, G**). The CCK-8 assay demonstrated that AGBL4 overexpression enhanced the proliferation ability of GBM cells (**Figures 4A, B**). Additionally, the colony formation assay revealed that AGBL4 overexpression led to an increased number of colonies (**Figures 4C–F**). Scratch assays indicated that the high expression of AGBL4 promoted the migration of GBM cells (**Figures 4G–J**). Furthermore, the Matrigel-transwell assays demonstrated a significant increase in invasion, with more cell visible in the fields of view compared to the controls (**Figures 4K–N**).

These findings highlight a critical role for AGBL4 in promoting the proliferation, migration, and invasion of GBM cells.

### 3.4 AGBL4 knockdown significantly reduces a range of classic factors associated with cancer-related pathways

**Figure 5:**
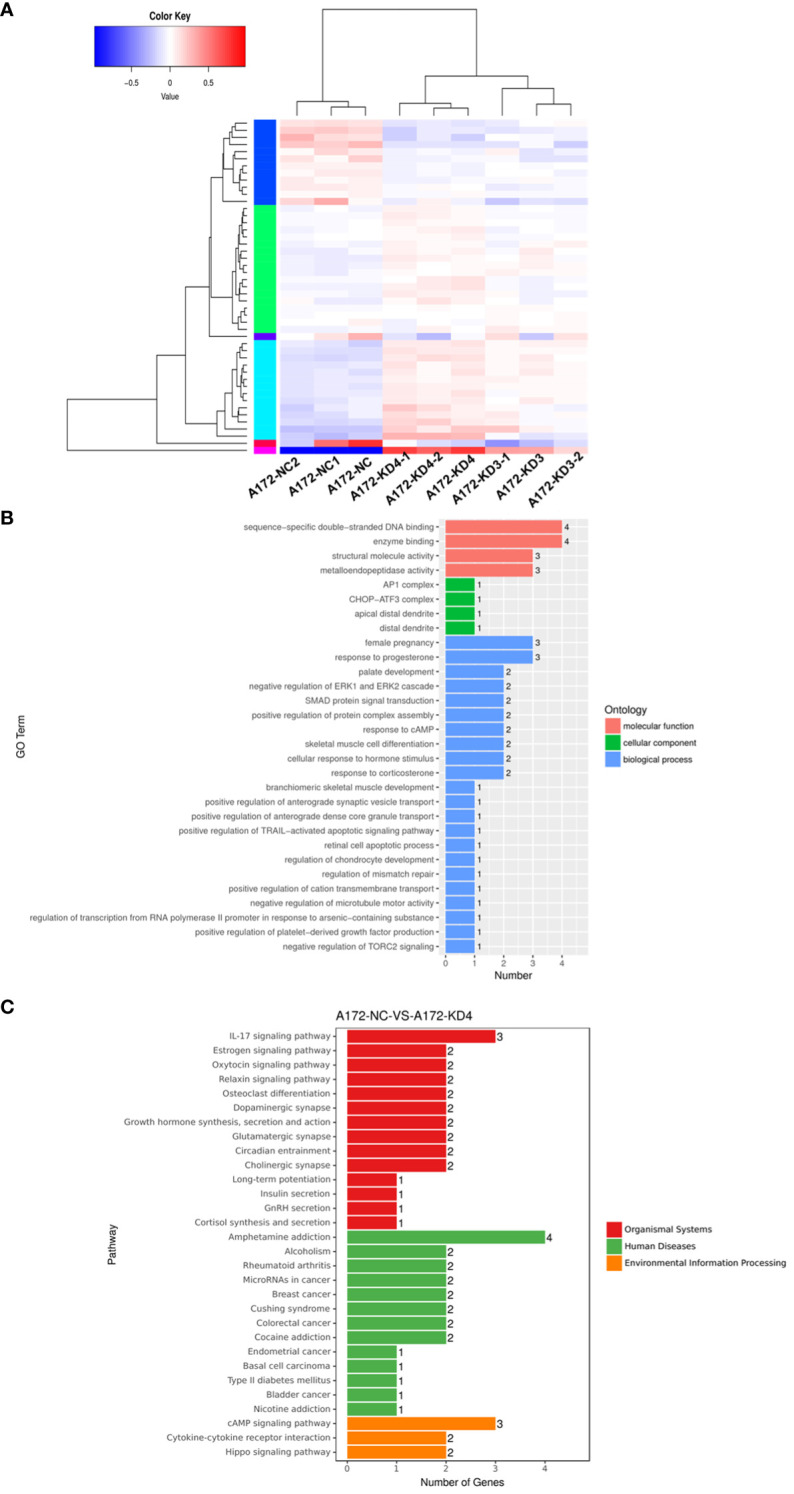
AGBL4 knockdown significantly reduces a range of classic factors associated with cancer-related pathways. **(A)** Heatmap showing differential gene expression between NC and AGBL4-KD in A172 cells. Each column represents a different sample, and each row represents a gene. Red indicates upregulated genes, blue indicates downregulated genes, and color intensity correlates with expression level. **(B)** GO annotations analysis of DEGs in A172 cells comparing NC and KD. The bar chart categorizes GO terms by biological processes, cellular components, and molecular functions, with the number of associated genes indicated. **(C)** KEGG analysis of DEGs in A172 cells after AGBL4-KD. The bar chart categorizes pathway terms by organismal systems, human diseases, and environmental information processing, with the number of genes involved in each pathway.

To elucidate the molecular mechanism of AGBL4 in GBM, we conducted transcriptome sequencing on A172 cells with or without AGBL4 knockdown. The heatmap revealed distinct differences and pairwise correlations in gene expression between the various GBM cell samples (**Figure 5A**). Analysis identified nearly 42 DEGs, with 30 up-regulated and 12 down-regulated (**Supplementary Figure 1**).

Bioinformatics analysis indicated that these DEGs were primarily involved in processes such as enzyme binding, positive regulation of protein complex assembly, positive regulation of TRAIL-activated apoptotic signaling pathway, and negative regulation of microtubule motor activity, according to GO annotations (**Figure 5B**). Furthermore, KEGG enrichment analysis suggested that AGBL4-associated DEGs might participate in pathways related to microRNAs in human cancer and contribute to the IL-17 signaling pathway, which is frequently recognized as a reference index to judge the malignancy of gliomas (**Figure 5C**). Based on these findings, we speculated that AGBL4-related DEGs might play significant roles in tumor progression within the central nervous system.

### 3.5 AGBL4 promotes GBM cell proliferation, migration, and invasion abilities via MMP-1

From the DEGs identified in our transcriptome analysis (**Supplementary Table 1**), eight candidate genes were selected based on fold change and prognostic correlation in TCGA database (**Table 1**). RT-PCR analysis revealed that among these candidates, MMP-1 exhibited the most significant differential expression (**Supplementary Figure 2**), identifying it as a target for further investigation to clarify the specific signaling pathway through which AGBL4 may promote GBM tumor progression.

**Table 1:** Candidate genes for downstream targets of AGBL4.

Candidate genes
CDCP1 PRUNE2 AXIN2 FRAS1
MMP-1 MIAT HSD17B6 SLITRK3

Microarray data revealed elevated MMP-1 expression in GBM tissues, categorized samples into high and low MMP-1 groups. Histologically, cells in the low MMP-1 group displayed uniform morphology, with regular arrangement and clear tissue structures, as confirmed by H&E staining. In contrast, the high MMP-1 group exhibited cells of varying sizes, irregular shapes, disorganized arrangement, significant nuclear atypia, and frequent mitosis, indicating a more aggressive cellular phenotype (**Supplementary Figure 3A**). Survival analysis displayed that patients with high MMP-1 expression had significantly shorter survival times than those with low expression (P=0.0149), indicating that MMP-1 levels are inversely correlated with GBM patient survival (**Supplementary Figure 3B**).

**Figure 6:**
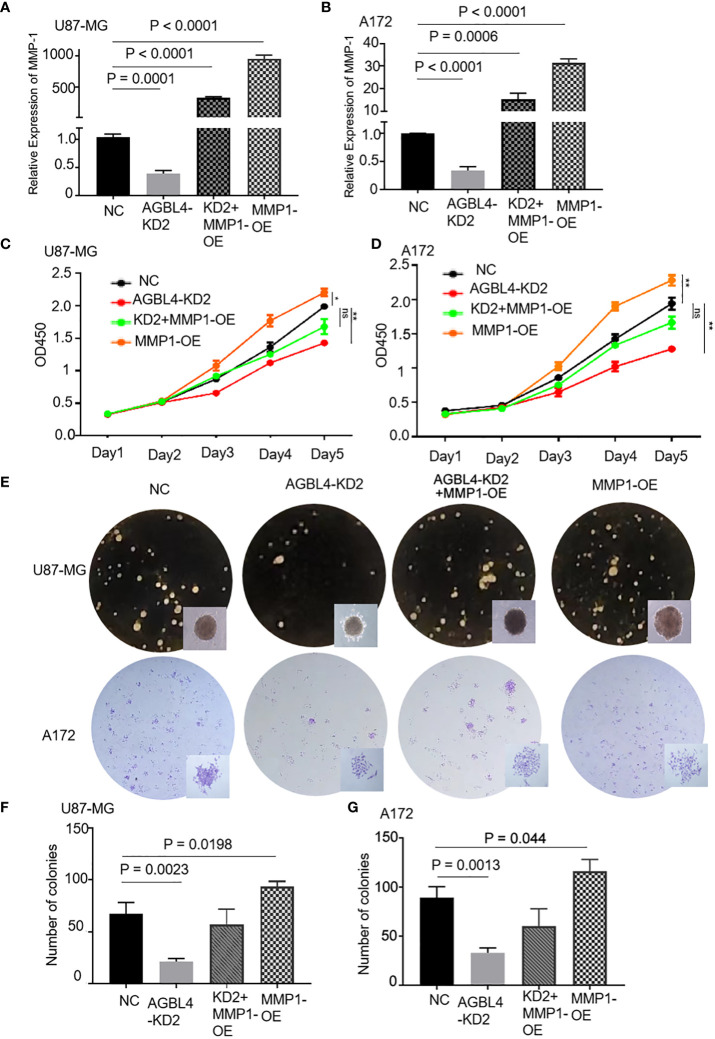
AGBL4 promotes GBM via MMP-1 and high expression of MMP-1 improves GBM cell proliferation abilities. **(A, B)** Relative expression levels of MMP-1 in NC, AGBL4-KD2, AGBL4-KD2+MMP1-OE, and MMP1-OE A172 and U87-MG cells. **(C, D)** Proliferation abilities of NC, AGBL4-KD2, AGBL4-KD2+MMP1-OE, and MMP1-OE A172 and U87-MG cells. **(E–G)** Number of formed colonies of NC, AGBL4-KD2, AGBL4-KD2+MMP1-OE, and MMP1-OE A172 and U87-MG cells.

**Figure 7:**
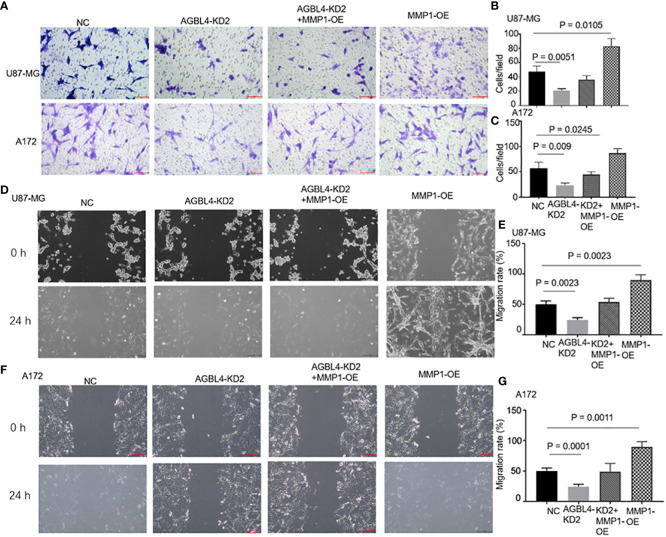
AGBL4 promotes GBM via MMP-1 and high expression of MMP-1 improves GBM cells migration and invasion abilities. **(A–C)** The numbers of invading cells in random fields of NC, AGBL4-KD2, AGBL4-KD2+MMP1-OE, and MMP1-OE A172 and U87-MG cells. **(D–G)** Migration levels of NC, AGBL4-KD2, AGBL4-KD2+MMP1-OE, and MMP1-OE A172 and U87-MG cells.

RT-PCR confirmed that compared to the U87-MG negative control (U87MG-NC group), knocking down AGBL4 (U87MG-AGBL4-KD2 group) significantly reduced the expression of MMP-1. Overexpressing MMP-1 in the AGBL4 knockdown cells (U87MG-AGBL4-KD2+MMP1-OE group) restored MMP-1 expression levels to those comparable with the control group (**Figures 6A, B**). Overexpression of MMP-1 on the basis of AGBL4 knockdown could counteract the inhibitory effect of AGBL4-decrease on GBM cells, which was manifested as the improvement of the proliferation capacity of AGBL4-knockdown U87-MG and A172 cells after complementing MMP-1 in CCK-8 assay (**Figures 6C, D**). Colony formation assays further supported this trend. Colony formation assays further supported this trend, with the MMP1-OE group demonstrating the strongest ability to form colonies. The AGBL4-KD2+MMP1-OE group’s colony-forming capacity was comparable to the NC. The AGBL4-KD2 group had the least robust colony-forming ability, reinforcing the significant role of MMP-1 in GBM cell proliferation (**Figures 6E–G**). The Matrigel-transwell and scratch assays indicated that, the MMP1-OE group exhibited the highest levels of invation and migration, followed by the AGBL4-KD2+MMP1-OE group, which displayed similar levels to the NC gtoup. Both of these groups exhibited enhanced capabilities compared to the AGBL4-KD2 group, which showed the lowest levels of invasion and migration (**Figure 7**).

### 3.6 Inhibition of AGBL4 suppresses GBM progression and prolongs survival via MMP-1 in animal models

**Figure 8:**
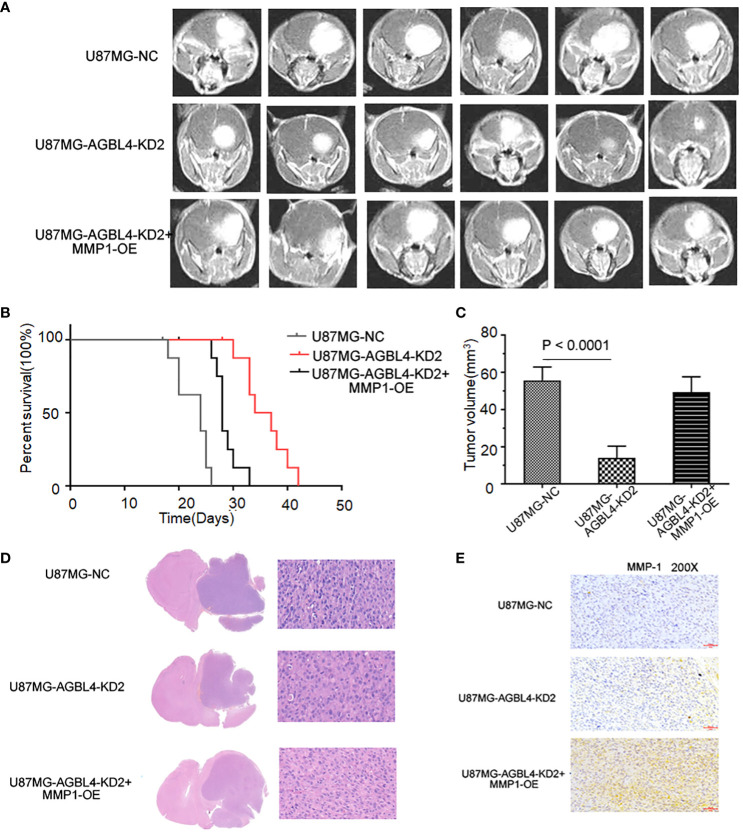
Inhibition of AGBL4 suppresses GBM progression and prolongs survival time in animal models. **(A)** MRI images showing the growth of intracranial tumors in mice implanted with U87-MG cells that are either NC, AGBL4-KD2, or AGBL4-KD2+MMP1-OE. **(B)** Kaplan-Meier survival curves of mice implanted with U87 cells that are either NC, AGBL4-KD2, or AGBL4-KD2+MMP1-OE, indicating the survival rate over time. **(C)** The quantification of tumor volumes of mice implanted with U87-MG cells that are either NC, AGBL4-KD2, or AGBL4-KD2+MMP1-OE, measured from the MRI images, with statistical significance indicated by P<0.0001. **(D)** H&E staining of intracranial tissues of the nude mice with U87MG-NC, U87MG-AGBL4-KD2, and U87MG-AGBL4-KD2+MMP1-OE. **(E)** Immunohistochemistry of intracranial tissues of the nude mice with U87MG-NC, U87MG-AGBL4-KD2, and U87MG-AGBL4-KD2+MMP1-OE (Magnification: 200×).

To determine the effect of AGBL4 and MMP-1 in GBM *in vivo*, we injected U87MG-NC, U87MG-AGBL4-KD2 and U87MG-AGBL4-KD2+MMP1-OE cells into nude mice (n=6). After intracranial tumor implantation, the mice were monitored every 3 days for behavioral changes and weight loss. On approximately day 15, MRI was performed to assess tumor growth when clinical symptoms were noted. The MRI data revealed that the U87MG-AGBL4-KD2 group exhibited significantly slower tumor growth compared to the U87MG-NC group. Conversely, the U87MG-AGBL4-KD2+MMP1-OE group showed accelerated tumor progression relative to the U87MG-AGBL4-KD group (**Figures 8A, C**). Survival analysis indicated that the U87MG-AGBL4-KD2 mice had the longest survival time, followed by the U87MG-AGBL4-KD2+MMP1-OE and U87MG-NC groups (**Figure 8B**). H&E staining of nude mice’s brain tissues displayed that there were more mitotic figures in U87MG-NC mice, followed by U87MG-AGBL4-KD2+MMP1-OE mice, while the morphology of cells from AGBL4-KD2 mice was relatively less irregular as well as fewer mitotic figures (**Figure 8D**). The protein content of tumor cells in the three group of nude mice differed from one another, that is, the degree of tumor progression was quite different. The proliferation level and the malignancy degree of U87MG-NC mice and U87MG-AGBL4-KD2+MMP1-OE mice were both higher than AGBL4-KD2 mice (**Figure 8E**).

### 3.7 AGBL4-MMP-1 axis is associated with inflammatory response pathways in GBM

**Figure 9:**
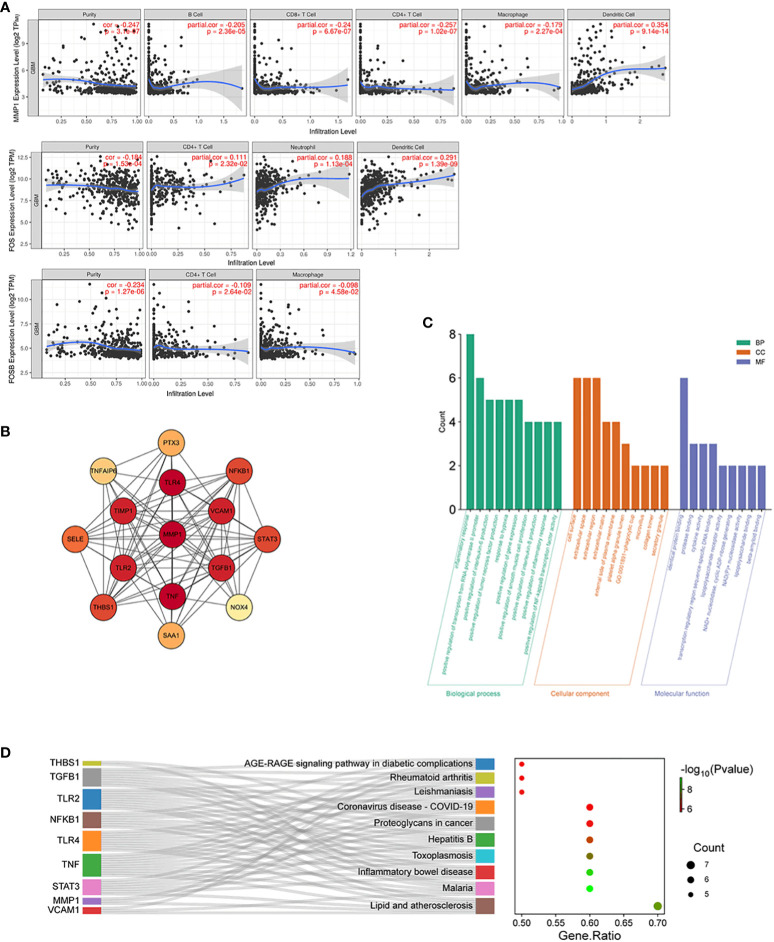
Immune cell correlation and molecular interaction analysis in GBM. **(A)** Scatter plots illustrating correlation between MMP-1, FOS, and FOSB gene expression and the infiltration levels of immune cells in GBM, adjusted for tumor purity, P < 0.05. **(B)** PPI network of MMP-1 with associated genes from the Inflammatory Response annotation cluster (GO:0006954), consisting of 15 nodes and 87 edges with MMP-1 centrally positioned. **(C)** GO annotations analysis for the top 10 hub genes from the PPI network. **(D)** KEGG pathway enrichment analysis for the top 10 hub genes from the PPI network, and the relationship between genes and pathways.

Enrichment analysis of AGBL4-related DEGs suggests that 3 genes, including MMP-1, FOS, and FOSB, are significantly concentrated in IL-17 signaling pathway. This may indicate that upregulated AGBL4, along with downstream MMP-1, could intervene in the progression of GBM by influencing key components within inflammation-related pathways. In the TIMER database, an immune cell correlation analysis of MMP-1, FOS, and FOSB revealed a negative correlation between MMP-1 gene expression and the infiltration levels of B cells, CD8+ T cells, CD4+ T cells, and macrophages, after purity adjustment. Conversely, a positive correlation with dendritric cell infiltration was observed. Meanwhile, FOS gene expression showed a positive correlation with the infiltration levels of CD4+ T cells, neutrophils, and dendritic cell infiltration. Besides, FOSB gene expression demonstrated a negative correlation with CD4+ T cell infiltration and macrophage infiltration levels (**Figure 9A**). These findings suggest that the expression levels of MMP-1, FOS, and FOSB are closely related to immune cell activity in GBM, hinting at the role of these genes, particularly MMP-1, in modulating the GBM immune microenvironment.

### 3.8 PPI network and correlation analysis of MMP-1 and inflammatory response genes

We then constructed an interaction network integrating MMP-1 with 737 genes from the Inflammatory Response annotation cluster (GO:0006954) of GO database to identify key molecules interacting with MMP-1, which resulted in a PPI network comprising 15 nodes and 87 edges (**Figure 9B**). Excavation of this network yielded the top 10 hub genes, which were then subjected to GO and KEGG enrichment analyses. The results, as shown in **Figures 9C, D**, revealed that these hub genes are predominantly localized to the cell surface, extracellular space, and extracellular region, and are involved in various inflammatory and immune regulatory processes such as the inflammatory response, positive regulation of transcription from RNA polymerase II promoter, and positive regulation of interleukin-6 production. KEGG pathway analysis also indicated significant enrichment in several pathways related to inflammation and immune responses. Collectively, these findings underscore the role of genes interacting with MMP-1 in regulating inflammatory responses, immune signal transduction, and cell proliferation, invasion, and migration, indirectly reflecting the importance of MMP-1 in maintaining tissue structure and signal transduction within the inflammatory and tumor microenvironment.

To further examine the correlation between MMP-1 expression levels and the expression of inflammatory response genes in GBM samples, we utilized data from TCGA database. The results indicated a moderate positive correlation between MMP-1 and several genes, including NFKB1, SELE, TGFB1, THBS1, TIMP1, and TNFAIP6. A weaker positive correlation was observed between MMP-1 and PTX3, STAT3, TLR2 (**Supplementary Figure 4**). These findings corroborate, at the expression level, the involvement of MMP-1 with these genes in certain biological processes or pathological mechanisms within GBM, particularly in pathways related to the inflammatory response.

### 3.9 Mutation profile and prognostic value of inflammatory response genes interacting with MMP-1

**Figure 10:**
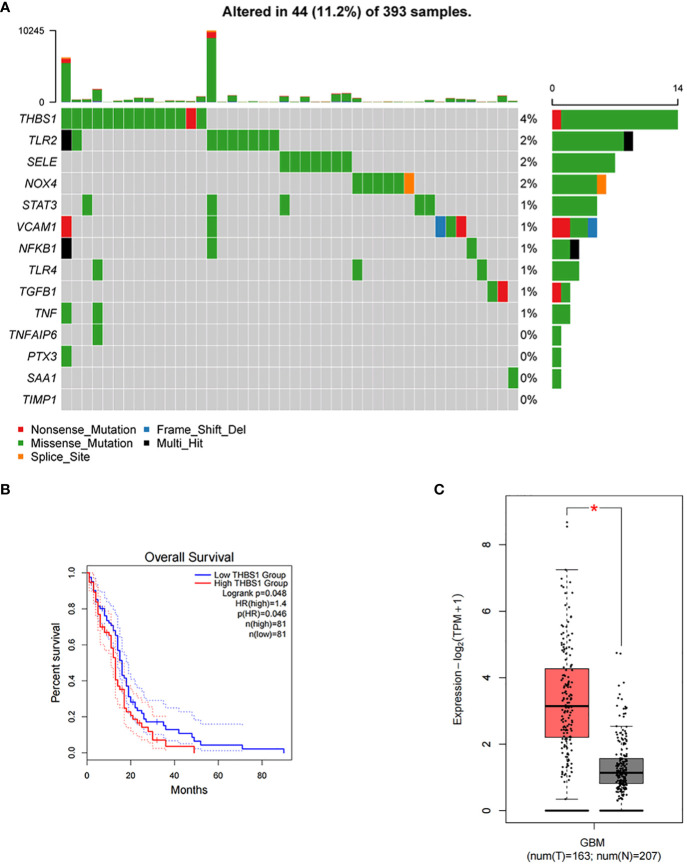
Mutational landscape of inflammatory response genes and their impact on survival in GBM. **(A)** A mutational landscape displaying the frequency and types of genetic alterations in inflammatory response genes across 393 GBM samples from TCGA with each row representing a gene, and each column a sample. Alterations, including nonsense mutations, frame shift deletions, missense mutations, multi-hit events, and splice site alterations, are color-coded. The graph on the right side indicates the percentage of samples with mutations in each gene, with the graph on the top showing the total number of mutations per sample. **(B)** Kaplan-Meier survival curves comparing overall survival between GBM patients with high and low expression of THBS1, P=0.046. **(C)** A box plot illustrating the differential expression of THBS1 between tumor (T) tissue samples in GBM patients from TCGA and normal (N) brain tissue samples from GTEx database, with the red asterisk denoting a statistically significant higher expression in the tumor samples.

**Figure 10A** presents the mutation profile of the 14 inflammatory response genes that interact with MMP-1 in GBM from TCGA database. It is observed that over 10% of the samples harbor mutations in at least one of the aforementioned genes, with THBS1 exhibiting the highest mutation frequency, nearing 4%. The predominant type of mutation found in most inflammation-related genes is missense mutation. VCAM1 harbors frame shift deletions, while THBS1, VCAM1, and TGFB1 contain nonsense mutations, and NOX4 shows splice site mutations. These mutation data provide insight into the functional roles of MMP-1 and associated inflammatory response genes in GBM, suggesting they may influence protein function through alterations in amino acid sequences, premature protein translation termination, protein inactivation, or changes in protein structure, thereby affecting inflammatory and immune responses and ultimately contributing to tumor progression. Bioinformatic analyses of these inflammatory response genes revealed that high expression levels of THBS1 correlate with a lower overall survival rate in GBM patients (**Figure 10B**), implying that THBS1 may be an adverse prognostic factor. **Figure 10C** reconfirms the expression levels of THBS1 in GBM from TCGA database compared to normal brain tissue in GTEx database, where THBS1 is significantly overexpressed in tumor tissues. These findings may signify a detrimental role of THBS1 in the pathological process of GBM, where its elevated expression reflects more aggressive biological characteristics of the tumor and provides direction for the development of future biomarkers.

Combining immune cell correlation analysis, PPI network construction, gene mutation profiling, and correlative studies, we can tentatively conclude that the interactions among AGBL4, MMP-1, and other inflammatory response genes, especially THBS1, may constitute a complex network in the pathological process of GBM. This network potentially regulates the tumor microenvironment, influencing tumor proliferation, invasion, migration, and patient survival. These findings highlight the potential of inflammation-related factors as focal points for future research, offering the possibility to further explore the precise mechanisms of these molecules and provide critical information for the development of novel therapeutic strategies.

## 4 Discussion

AGBL4, also named as cytosolic carboxypeptidase 6, is part of the family of enzymes that catalyze the deglutamylation of polyglutamate side chains on proteins such as tubulins and nucleosome assembly proteins (10). Polyglutamylation is a reversible post-translational protein modification and has been found playing a critical role in tubulin regulation as well as in cellular processes such as chromatin remodeling or hematopoiesis (10, 11). Besides, alterations in polyglutamylation levels have been associated with several pathologies, including neurodegenerative processes or cancer progression (12, 13). As a member of cytosolic carboxypeptidase family, although the role of AGBL4 in various cellular and pathological processes such as antiviral activity, immunomodulatory activity, and renal adenocarcinamo is documented (14–16), its function in central nervous system tumors, particularly GBM, has been less explored. Our study made an approach to the involvement of AGBL4 in GBM pathogenesis and its potential mechanism of action through the modulation of MMP-1.

Our finding indicate that elevated AGBL4 expression correlates with poor prognosis in GBM patients, which aligns with data from both TCGA and our tissue microarray experiments. The promotion of GBM cell proliferation, invasion, and migration by AGBL4 was substantiated through phenotypic experiments. Transcriptomic and bioinformatic analyses further revealed that AGBL4-realted DEGs were enriched in cancer-associated microRNA-related pathways and IL-17 signaling pathway, the latter being notably related to malignancy in central nervous system tumors. This suggests a possible link between AGBL4’s oncogenic effects and inflammatory pathways, highlighting its role in the tumor microenvironment’s immune responses.

The matrix metalloproteinase family, particularly MMP-1, known for its role in cleaving collagenous extracellular matrix (17), appears to be a critical downstream effector of AGBL4. Elevated MMP-1 expression is a hallmark of highly malignant gliomas and is implicated in enhancing tumor invasiveness and malignancy (18, 19). Pullen et al. demonstrated a regulatory pathway linking nitric oxide to high-grade glioma cell motility via MMP-1 (20). Anand et al. identified that EGFR regulates MMP-1 predominantly through the MAPK signaling pathway in GBM cells (21). Malik et al. found an association between the 2G/2G genotype and 2G allele of -1607 MMP-1 polymorphism and GBM occurrence (22). Additionally, increased MMP-1 and PAR1 expression correlates with higher histological malignancy and poorer clinical outcomes in gliomas (23). While much research has focused on MMP-1’s downstream mechanisms in gliomas, its upstream regulators remain underexplored, which is crucial for understanding glioma invasiveness.

Our study not only confirms the upregulation of MMP-1 in high-grade gliomas but also identifies AGBL4 as a novel upstream regulator of MMP-1. Existing studies on AGBL4 are relatively few and mainly focus on its role in cellular component (24), neurodegeneration (25), and immunomodulatory activities (16, 26). However, its implications in oncology, particularly in GBM, have been less explored. Our study marks a significant advancement by first identifying the differential expression of AGBL4 in GBM and verifying its negative correlation with patient survival through analysis of public databases and gene chips. This groundbreaking research links AGBL4 to the aggressive nature of central nervous system tumors at the molecular level for the first time. Further, our experimental findings underscore the critical role of AGBL4 in tumor biology, revealing that knocking down AGBL4 inhibits the proliferation, migration, and invasion of GBM cells, thertby highlighting its importance in tumor viability and progression. Importantly, this research not only pioneers the investigation of the interaction of AGBL4 with GBM, but also introduces the novel concept that AGBL4 may contribute to GBM in an MMP-1-dependent manner.

In addition, the interaction between AGBL4 and MMP-1 highlights a potential connection to the inflammatory processes within the tumor microenvironment of GBM. The upregulation of MMP-1, mediated by AGBL4, may not only promote tumor invasiveness through structural modifications but could also exacerbate inflammation, thereby creating a more conducive environment for tumor growth and spread. Our data indicates that the expression levels of MMP-1, FOS, and FOSB are closely related to immune cell activity in GBM, suggesting their pivotal roles in modulating the GBM immune microenvironment.

Our constructed PPI network, integrating MMP-1 with genes from the Inflammatory Response cluster of the GO database, identified key molecules that interact with MMP-1. These interacting genes are primarily involved in inflammatory response, positive regulation of transcription from RNA polymerase II promoter, and positive regulation of interleukin-6 production, indirectly reflecting the importance of MMP-1 in maintaining tissue structure and signal transduction within the inflammatory and tumor microenvironment.

Further analysis from TCGA database on the correlation between MMP-1 expression levels and the expression of inflammatory response genes in GBM samples showed a moderate positive correlation between MMP-1 and several genes, exemplified by THBS1, confirming the involvement of AGBL4-MMP-1 axis in GBM-related inflammatory pathways.

However, understanding the molecular pathogenesis of GBM remains a challenge. It is speculated that AGBL4 and MMP-1 may contribute to the occurrence, development, and spread of GBM, but the specific mechanism and interactions between AGBL4 and MMP-1 still require further investigation.

## 5 Conclusion

**Figure 11:**
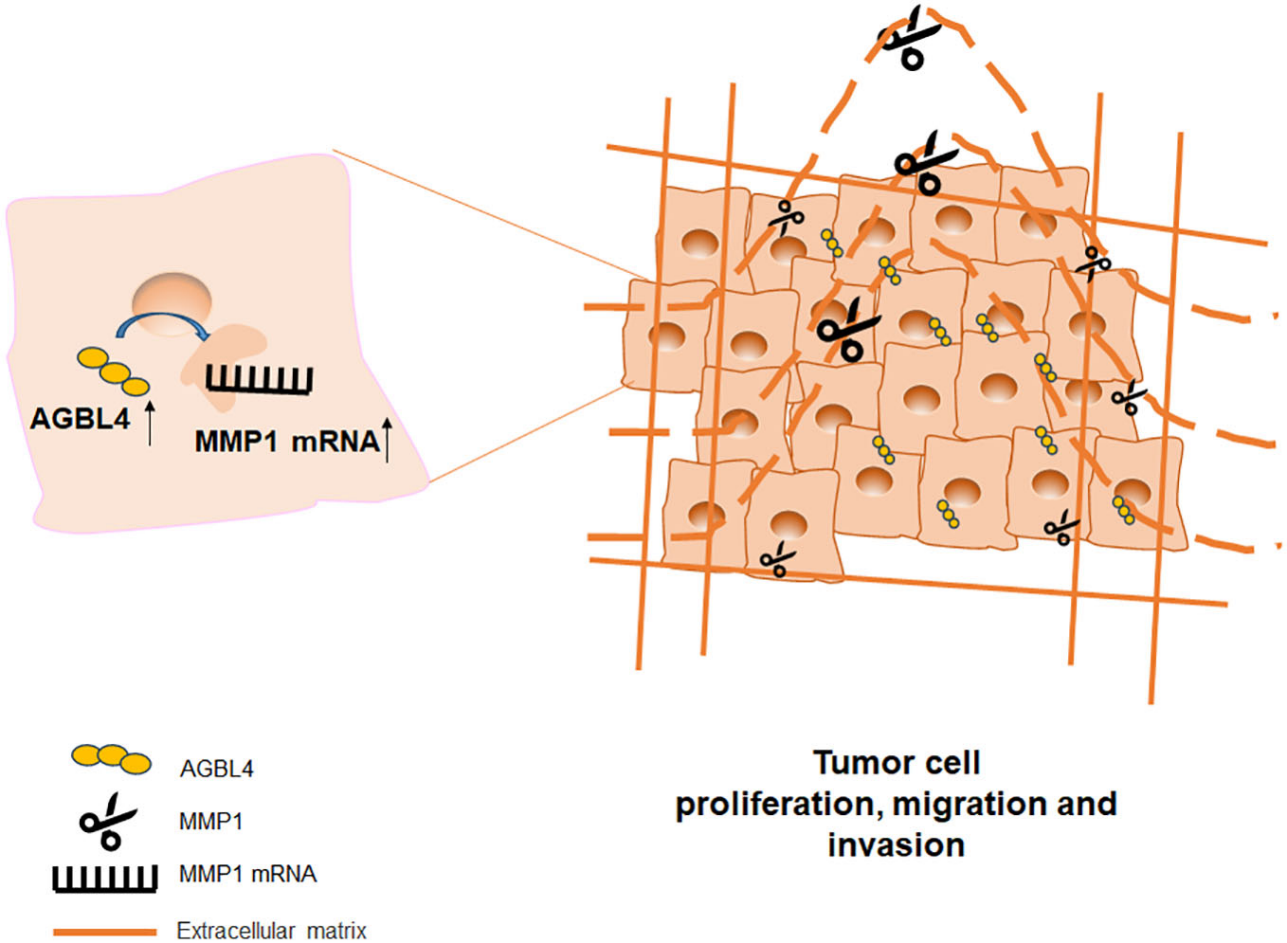
Model for the mechanism of AGBL4 in GBM tumorigenesis.

In summary, this study demonstrates that AGBL4 expression in GBM is upregulated and links with poor prognosis of GBM patients by enhancing tumor cell proliferation, migration, and invasion. Our findings reveal a novel mechanistic pathway where AGBL4 enhances GBM malignancy primarily through modulation of MMP-1 expression, which in turn influences the inflammatory response pathways within the tumor microenvironment (**Figure 11**). The identification of AGBL4 and MMP-1 not only deepens our understanding of the molecular dynamics of GBM but also highlights their involvement in inflammatory processes that may contribute to tumor aggressiveness, suggesting the potential of AGBL4 and MMP-1 as strategic targets for gene-directed therapy, as well as advocating for the development of targeted inhibitors against these proteins as a promising new direction for therapeutic intervention in glioma treatment.

## Data availability statement

The datasets presented in this study can be found in online repositories. The names of the repository/repositories and accession number(s) can be found below: GSA-Human: HRA003631 (Genome Sequence Archive in the National Genomics Data Center, China National Center for Bioinformation / Beijing Institute of Genomics, Chinese Academy of Sciences, https://ngdc.cncb.ac.cn/gsa-human).

## Ethics statement

The studies involving humans were approved by the Ethical Committee of Changhai Hospital, Naval Medical University. The studies were conducted in accordance with the local legislation and institutional requirements. The participants provided their written informed consent to participate in this study. The animal study was approved by the Institutional Animal Care and Use Committee of Changhai Hospital, Naval Medical University. The study was conducted in accordance with the local legislation and institutional requirements.

## Author contributions

SZ: Writing – review & editing, Conceptualization, Data curation, Formal analysis, Investigation, Methodology, Writing – original draft, Visualization. LC: Visualization, Writing – review & editing, Data curation, Formal analysis, Investigation, Methodology, Software, Writing – original draft. YS: Writing – review & editing, Data curation, Formal analysis, Software, Supervision, Visualization, Writing – original draft. ZQ: Writing – review & editing, Data curation, Formal analysis, Investigation, Software, Writing – original draft, Resources. ZW: Software, Supervision, Visualization, Writing – review & editing, Validation. CC: Software, Supervision, Writing – review & editing, Validation. RL: Writing – review & editing, Formal analysis, Investigation. AZ: Software, Writing – review & editing, Formal analysis. JH: Validation, Writing – review & editing, Visualization. JM: Supervision, Writing – review & editing, Software. HW: Writing – review & editing, Conceptualization, Data curation, Project administration, Supervision, Validation, Visualization. JC: Conceptualization, Funding acquisition, Methodology, Project administration, Resources, Supervision, Validation, Writing – review & editing.
