# Supplementary material for: AGBL4 promotes malignant progression of glioblastoma via modulation of MMP-1 and inflammatory pathways
Source: Front Immunol. 2024 Jun 28;15:1420182. doi: 10.3389/fimmu.2024.1420182 (PMC11246717; doi:10.3389/fimmu.2024.1420182)
Supplement: Supplementary file 1 [file DataSheet_1.docx]

Supplementary Materials for

**AGBL4 Promotes Malignant Progression of Glioblastoma via Modulation of MMP-1 and Inflammatory Pathways**

**Manuscript ID: 1420182**

**Authors:** Shuai Zhang^1†^, Lilin Cheng^1†^, Yandong Su^1†^, Zhongrun Qian^2†^, Zhen Wang^1^, Chao Chen^1^, Rong Li^1^, Aikang Zhang^1^, Jiawei He^1^, Jiangxin Mao^1^, Hongxiang Wang^1^*, Juxiang Chen^1^*

Correspondenct to: wanghongxiang27@smmu.edu.cn or jxchenchhs@163.com

**Supplementary Methods**

**Hematoxylin-Eosin (H&E) Staining**

Tissue sections were fixed, embedded in paraffin, and cut into 5 µm thick slices. Sections were then deparaffinized, rehydrated, and stained with Harris hematoxylin followed by eosin Y. After staining, sections were dehydrated, cleared, and mounted. The stained tissues were analyzed under a light microscope to evaluate histopathological features.

**Functional Enrichment Analysis**

Functional enrichment analysis was performed using the Database for Annotation, Visualization, and Integrated Discovery (DAVID, v2021) for Gene Ontology (GO) and Kyoto Encyclopedia of Genes and Genomes (KEGG) pathway analyses. The Tumor IMmune Estimation Resource (TIMER) database was used to analyze immune cell infiltration levels and their correlation with gene expression. The STRING database (version 12.0) was utilized for protein-protein interaction (PPI) networks, The Ctyoscape software (version 3.10.2) was used for data visualization and hub gene mining.


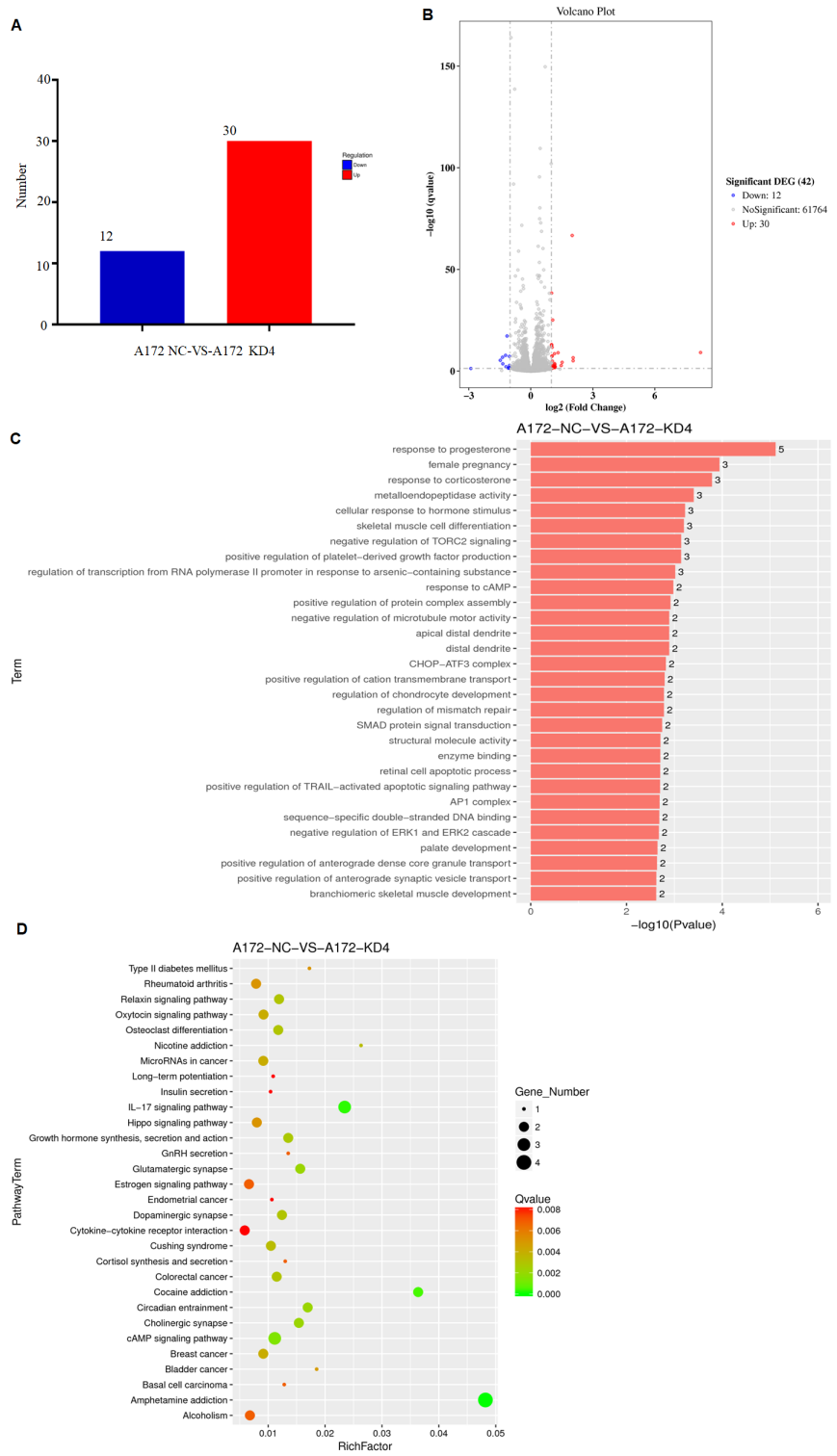


**Supplementary Figure 1 Bioinformatics analysis of DEGs**

**A** Results of differentially expressed genes analysis of A172 and A172-AGBL4-KD4. **B** Volcano map of DEGs of A172 and A172-AGBL4-KD4. **C** GO enrichment analysis of DEGs of A172 and A172-AGBL4-KD4. **D** KEGG enrichment analysis of A172 and A172-AGBL4-KD4.


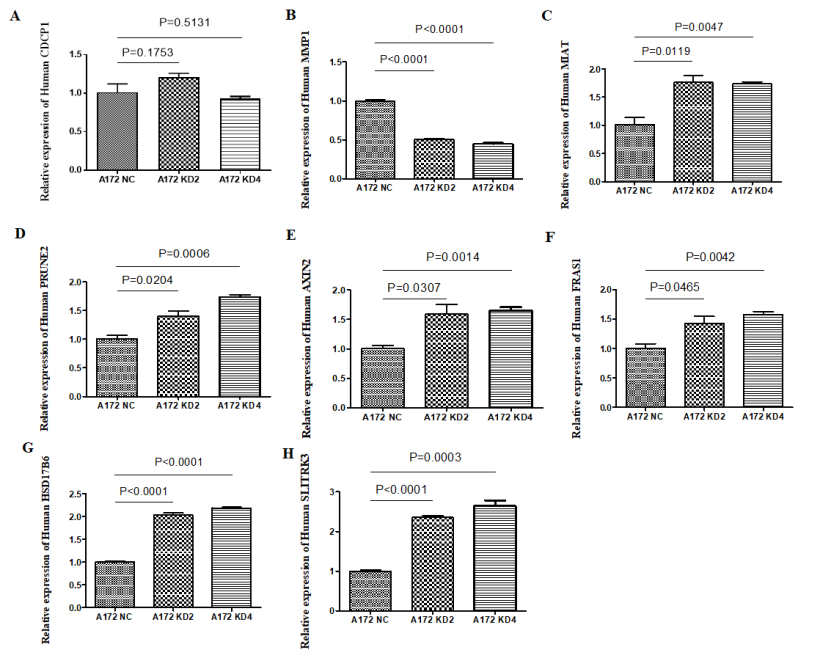


**Supplementary Figure 2 Relative expression levels of candidate genes**

**A-H** The relative expression levels of CDCP1 (**A**), MMP-1 (**B**), MIAT (**C**), PRUNE2 (**D**), AXIN2 (**E**), FRAS1 (**F**), HSD17B6 (**G**) and SLITRK3 (**H**).


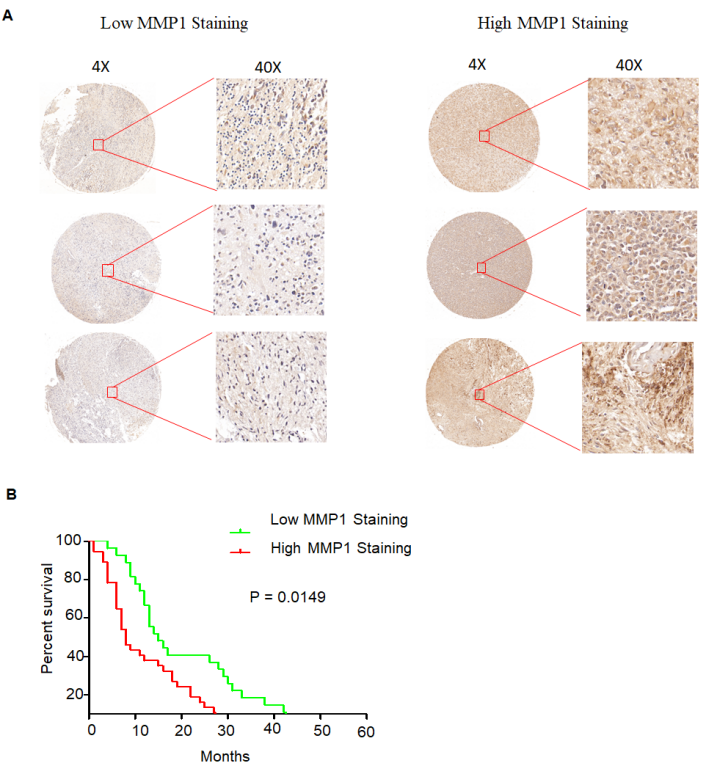


**Supplementary Figure 3 MMP-1 is highly expressed in GBM and links with poor prognosis**

**A** Hematoxylin-eosin staining of GBM tissues with low and high MMP-1. **B** Survival curve of GBM patients with low and high MMP-1 staining, P=0.0149.





**Supplementary Figure 4 The correlation expression level between MMP-1 and inflammatory response genes in GBM samples**

**A-I** The correlation expression levels between MMP-1 and NFKB1 (**A**), PTX3 (**B**), SELE (**C**), STAT3 (**D**), TGFB1 (**E**), THBS1 (**F**), TIMP1 (**G**), TLR2 (**H**) and TNFAIP6 (**I**).

**Supplementary Table 1** All the DEGs for downstream targets of AGBL4
